# An open-label phase 2 trial to assess the efficacy, safety and pharmacokinetics of lanthanum carbonate in hyperphosphatemic children and adolescents with chronic kidney disease undergoing dialysis

**DOI:** 10.1186/s12882-022-02688-9

**Published:** 2022-03-02

**Authors:** Anna Wasilewska, Rose Ann Murray, Aimee Sundberg, Sharif Uddin, Heinrich Achenbach, Aleksey Shavkin, Tamás Szabó, Andrea Vergani, Obi Umeh

**Affiliations:** 1 Department of Pediatrics and Nephrology, Faculty of Medicine, Medical University of Bialystok, University Children’s Clinical Hospital of Bialystok, Waszyngtona, Bialystok, Poland; 2 Shire Human Genetic Therapies, Inc., a Takeda Company, Cambridge, MA USA; 3 Takeda Pharmaceuticals USA, Inc., Lexington, MA USA; 4 Shire Human Genetic Therapies, Inc., a Takeda Company, Zug, Switzerland; 5 Saint Petersburg State Budgetary Healthcare Institution, Children’s City Multidisciplinary Clinical Specialized Center of High Medical Technologies, Saint Petersburg, Russia; 6 Department of Pediatrics, Faculty of Medicine, University of Debrecen, Debrecen, Hungary

**Keywords:** Chronic kidney disease, End-stage renal disease, Lanthanum carbonate, Pediatric, Pharmacokinetics

## Abstract

**Background:**

This study assessed the efficacy, tolerability and pharmacokinetics (PK) of lanthanum carbonate (LC) in hyperphosphatemic children and adolescents with chronic kidney disease (CKD) undergoing dialysis.

**Methods:**

This was a three-part, multicenter, open-label study of LC (oral powder formulation) in patients 10 to < 18 years old with CKD undergoing dialysis. In part 1, the single-dose PK of LC (500 mg, ≤12 years old; 1000 mg, > 12 years old) were summarized. In part 2, patients received calcium carbonate (CC [chewable tablet formulation]) (1500–6500 mg [total daily dose]) followed by LC (powder formulation) (1500–3000 mg [total daily dose]), or LC only (1500–3000 mg [total daily dose]), each for 8 weeks. During part 3, patients received LC (1500–3000 mg [total daily dose]) for up to 6 months. The primary efficacy endpoint was the proportion of LC-treated patients achieving serum phosphorus control after 8 weeks during parts 2 and/or 3, defined as: ≤1.94 mmol/L, < 12 years old; ≤1.78 mmol/L, ≥12 years old. Secondary efficacy endpoints included: the proportion of patients who achieved serum phosphorus control after 8 weeks of treatment with CC followed by 8 weeks of treatment with LC (with a washout period between treatments). The safety of LC and CC was also evaluated.

**Results:**

In part 1, 20 patients received a single dose of LC. In part 2, 53 and 51 patients were treated with CC and LC for 8 weeks, respectively. During part 3, 42 patients received LC for up to 6 months. Most patients were white and male. For the primary efficacy endpoint, 50% (17/34) of patients who received LC for 8 weeks during parts 2 and/or 3 achieved serum phosphorus control. After 8 weeks of treatment with CC, 58.8% of patients achieved serum phosphorus control; after a subsequent washout period and 8 weeks of treatment with LC, 70.6% of patients achieved serum phosphorus control. T_max_ and t_1/2_ occurred within 3–8 h and ~ 19 h, respectively; however, variability was observed. LC and CC were generally well tolerated.

**Conclusions:**

These data support the use of LC to manage hyperphosphatemia in pediatric patients with CKD undergoing dialysis.

**Trial registration:**

ClinicalTrials.gov identifier: NCT01696279; EudraCT identifier: 2012–000171-17.

Date of registration: 01/10/2012.

**Supplementary Information:**

The online version contains supplementary material available at 10.1186/s12882-022-02688-9.

## Background

Chronic kidney disease (CKD) leads to a progressive decline in the kidney’s ability to excrete phosphate, and is accompanied by significant disruption to mineral metabolism [1–3]. The most advanced stage of CKD (stage 5) is characterized by a glomerular filtration rate of less than 15 mL/min/1.73 m^2^, and is classified as renal failure [4]. Hyperphosphatemia is a common clinical manifestation of advanced CKD [3]. Most patients with stage 5 CKD have serum phosphorus levels that exceed the normal range of 0.8–1.5 mmol/L, and dialysis is often insufficient to rectify this imbalance [5]. Hyperphosphatemia can result in severe pathophysiological consequences, such as an increased risk of cardiovascular events [6], renal osteodystrophy [7, 8], hyperparathyroidism [6] and all-cause mortality [5].

Control of serum phosphorus levels is fundamental to the management of CKD-related health risks [3]. The National Kidney Disease Outcomes Quality Initiative (KDOQI) guidelines provide target serum phosphorus levels for patients with CKD. These are age-specific and, at the time of study design, were defined as ≤1.94 mmol/L for patients younger than 12 years old and ≤ 1.78 mmol/L for patients between 12 and 18 years old [10–12]. For most patients, hyperphosphatemia is managed using phosphate-binding agents. Although phosphate binders are effective in combination with dietary phosphorus restriction and dialysis, their use is associated with a number of challenges [9, 13, 14]. Large doses of calcium-based phosphate binders, such as calcium carbonate (CC), are usually required to achieve sufficient control of phosphorus levels, which can cause hypercalcemia and vascular calcification [14, 15]. Aluminum-based phosphate binders have been linked to neurological and bone diseases [11], anemia and dementia [14]. Iron-based phosphate binders, such as ferric citrate, have been associated with increased serum ferritin and transferrin saturation [16]. Sevelamer carbonate has low phosphate-binding affinity and high off-target effects [17]. These include reducing serum low-density lipoprotein cholesterol, which is indicative of vascular calcification, and increasing fetuin-A concentrations.

The calcium-free phosphate binder lanthanum carbonate (LC) is indicated for the treatment of hyperphosphatemia in adults with end-stage renal disease (ESRD) [18]. The efficacy of LC, quantified by a reduction of serum phosphorus to KDOQI target levels, has been shown in adults at doses in the range of 750–3000 mg/day [19, 20]. The phosphate-lowering effect and tolerability of LC are similar to those of other available therapies, including calcium- and aluminum-based phosphate binders and sevelamer hydrochloride [21]. Furthermore, the excretion of LC is predominantly via non-renal routes, which is an important characteristic for drugs indicated for patients with impaired renal function [22].

Although LC has been associated with a greater likelihood of achieving serum phosphorus control in patients with ESRD, [23] a phase 1 study in healthy adult male participants reported that the bioavailability of LC (chewable tablet formulation) after a single oral dose was low (0.00127%) [24]. A 10-year safety analysis showed that LC (chewable tablet and oral powder formulations) was generally well tolerated, and that there was no evidence LC was associated with adverse safety outcomes in adults after more than 850,000 person-years of global patient exposure [22]. All-cause mortality and bone fracture rates in patients treated with LC were shown to be similar to those in patients treated with other phosphate binders.

In pediatric patients, hyperphosphatemia is preferentially managed by restricting dietary phosphorus intake; however, calcium-free phosphate binders such as sevelamer hydrochloride are widely used in patients with persistent electrolyte imbalance [25]. To date, there have been a limited number of clinical studies of phosphate binders conducted in pediatric populations [26–28]. These studies focused on calcium carbonate, calcium acetate and sevelamer hydrochloride, and showed efficacy in achieving serum phosphorus control in pediatric patients with CKD. The aim of this study was therefore to assess the efficacy, safety and pharmacokinetics (PK) of LC in hyperphosphatemic children and adolescents with CKD undergoing dialysis.

## Methods

### Study design and population

This was a three-part, open-label, phase 2, multicenter trial (ClinicalTrials.gov identifier: NCT01696279/EudraCT identifier: 2012–000171-17; date of registration: 01/10/2012) to assess the efficacy, safety and tolerability of 8 weeks to 8 months of treatment with LC (oral powder formulation) and 8 weeks of treatment with CC (chewable tablet formulation) in hyperphosphatemic children and adolescents, 6 months to younger than 18 years old, with CKD undergoing hemodialysis or continuous ambulatory peritoneal dialysis. The age criteria were later amended to include patients 10 years to younger than 18 years old owing to difficulties with the recruitment of younger patients.

According to the amended inclusion criteria, patients: were 10 years to younger than 18 years old at the time of consent; were male patients or non-pregnant, non-lactating female patients in compliance with contraceptive requirements; had a diagnosis of CKD and were undergoing dialysis, requiring treatment for hyperphosphatemia with a phosphate binder; and had serum phosphorus levels above age-specific KDOQI targets, after a washout period, of at least 1.94 mmol/L (patients < 12 years old) and at least 1.78 mmol/L (patients ≥12 years old), as per guidelines in effect at the time of the study design [10]. Exclusion criteria included: current or recurrent disease other than CKD or ESRD (e.g. cardiovascular, liver, unstable/uncontrolled gastrointestinal, malignant); history of a relevant physical or psychiatric medical disorder; inability to eat semi-solid foods or receiving total enteral alimentation; known or suspected intolerance or hypersensitivity to the study drug; history of alcohol or substance abuse; current use of any medication that could affect the condition being studied, interact with the study drug, or disrupt any clinical or laboratory assessment; weight and age outside criteria for blood sample volume limits; and use of another investigational product in the 30 days before the first dose of the study drug.

#### Study design (screening, washout and part 1) – single oral dose of LC for PK analysis

**Fig. 1:**
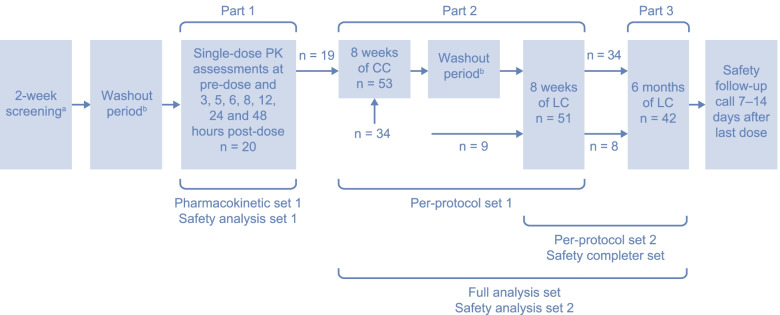
Overview of the study design. ^a ^Screening was only required once, either before part 1, if participating, or before part 2 of the study. ^b ^The washout period was up to 3 weeks and was only required if serum phosphorus levels were below age-specific KDOQI targets *CC* calcium carbonate, *KDOQI* Kidney Disease Outcomes Quality Initiative, *LC* lanthanum carbonate, *PK* pharmacokinetic

The first stage of the study comprised a 2-week screening period, after which eligible patients entered a washout period of up to 3 weeks if their serum phosphorus levels were below the age-specific KDOQI targets (Fig. 1) [10]. Patients were then assessed for inclusion based on their serum phosphorus levels. In part 1, patients received a single dose of LC (oral powder formulation) after food in the morning, the day after dialysis treatment, with differing doses based on age (500 mg, patients ≤12 years old; 1000 mg, patients > 12 years old). Blood samples were taken for PK analyses before administration of LC and at 3, 5, 6, 8, 12, 24 and 48 h after administration.

#### Study design (part 2) – 8-week treatment periods with CC and/or LC

Patients from part 1 of the study could enter part 2 immediately after the completion of single-dose PK assessments; patients could also enroll directly into part 2 of the study. The screening and washout were only required for patients who did not participate in part 1. Patients were treated with either CC (chewable tablet formulation) for 8 weeks followed by LC for 8 weeks, or only LC for 8 weeks (Fig. 1). CC dosing was based on either the dose regimen taken by patients before study commencement or standard clinical practices for new patients. The starting total daily dose of LC was 1500 mg for all patients, based on the minimally effective dose established during a long-term open-label study of LC in adults (SPD405–307) [20]. LC (powder formulation) was mixed with food three times daily; if patients only had two meals per day, the dose was split equally across the two meals, with a single oral dose not exceeding 1000 mg. Daily doses could be adjusted at the discretion of the investigator to a maximum of 6500 mg for CC and 3000 mg for LC, until serum phosphorus control was achieved and maintained until the end of each respective treatment period. A washout period of up to 3 weeks was carried out between the CC and LC treatment periods until serum phosphorus levels were above age-specific KDOQI targets. In part 2, serum phosphorus, calcium and calcium-phosphorus product levels were measured at weeks 2, 4 and 6 of treatment and at the end of each 8-week treatment period. Local laboratory tests carried out at weeks 2, 4 and 6 of treatment during part 2 were used for titration purposes and were not evaluated for pre-specified efficacy analyses.

#### Study design (part 3 and follow-up) – 6-month extension study of LC treatment

Patients who completed part 2 of the study (or who discontinued CC treatment owing to hypercalcemia) were eligible to enter part 3 of the study immediately. In this part, patients were treated with LC for up to an additional 6 months, with serum phosphorus, calcium and calcium-phosphorus product levels measured every 4 weeks up to week 32 of treatment (Fig. 1). When appropriate, and at the discretion of the investigator, daily LC doses were adjusted up to a maximum dose of 3000 mg to maintain serum phosphorus control. A safety follow-up telephone call was scheduled between 7 and 14 days after the last dose of LC. Patients who discontinued phosphate binder treatment owing to kidney transplantation had their safety follow-up telephone call 30 ± 7 days after the last dose of LC.

### Study endpoints

The primary efficacy endpoint was the proportion of patients achieving age-specific KDOQI serum phosphorus target levels after receiving LC for 8 weeks during part 2 and/or part 3 of the study. This included patients who received CC only during part 2 but received at least 8 weeks of LC during part 3. For efficacy analyses of patients during part 2 and/or part 3 of the study, patients were only counted once. Secondary endpoints included the proportion of patients achieving age-specific KDOQI serum phosphorus target levels after 8 weeks of CC treatment followed by 8 weeks of LC treatment (with a washout period between treatments), and the proportion of patients with serum phosphorus levels at or below age-specific KDOQI targets by week of treatment with LC up to 32 weeks, during part 2 and/or part 3. The changes from baseline in serum phosphorus, calcium and calcium-phosphorus product levels after 8 weeks of treatment with LC, 8 weeks of CC treatment followed by 8 weeks of LC treatment (with a washout period between treatments) during part 2, and up to 8 months of total treatment with LC during part 2 and/or part 3 were also measured as secondary efficacy endpoints. Other secondary efficacy endpoints were changes from baseline in height and weight of patients after each 8-week treatment regimen (CC or LC), and changes from baseline in biochemical bone markers during part 2 and/or part 3: bone alkaline phosphatase, osteocalcin, tartrate-resistant acid phosphatase, fibroblast growth factor-23, parathyroid hormone, sclerostin and fetuin-A. Biochemical bone markers were measured owing to their suspected link to bone metabolism and cardiovascular calcification, morbidity and mortality in patients with CKD [29, 30].

The PK profile of LC after a single oral dose was described, and the safety profiles of CC over 8 weeks and LC over up to 8 months were also summarized.

Study drug compliance was also monitored for all patients during part 2 and part 3 of the study and was defined as the number of sachets or tablets taken by a patient divided by the total number of sachets or tablets expected to be taken, multiplied by 100.

### PK analyses

In part 1, serial blood samples were taken from patients before LC administration and at 3, 5, 6, 8, 12, 24 and 48 h after administration. PK analyses were performed for patients who received the single oral dose of LC in part 1 and had at least one measurable plasma concentration of LC (defined as ≥0.05 ng/mL) during the 48 h after administration.

PK parameters measured were: area under the curve (AUC) extrapolated to infinity (AUC_0–inf_), calculated using the last measurable LC concentration; AUC from the time of dosing to 48 h after dosing (AUC_0–48_); AUC from the time of dosing to the last measurable concentration (AUC_last_); total body clearance for extravascular administration divided by the fraction of dose absorbed (CL/F); maximum observable plasma concentration (C_max_); time of maximum observed plasma concentration (t_max_); terminal half-life (t_1/2_); volume of distribution associated with the terminal slope after extravascular administration divided by the fraction of dose absorbed (V_z_/F); and the first-order rate constant associated with the terminal (log-linear) portion of the curve (λ_z_).

### Safety assessments

Safety assessments included: monitoring adverse events, vital signs and electrocardiogram variables; weight and height assessments; physical examinations; and clinical laboratory tests (chemistry and hematology, and biochemical bone markers).

### Statistical analysis

#### Determination of sample size

Part 1 of this study: in a previous phase 1 study of LC (LAM-IV-111) [31], which compared the single-dose PK of LC (1000 mg) in healthy volunteers and patients undergoing dialysis, the mean (standard deviation [SD]) AUC_0–t_ and C_max_ were 3.10 (2.89) h·ng/mL and 0.30 (0.18) h·ng/mL, respectively. Given these observations, it was determined that the required sample size to achieve 80% assurance, when the two-sided 95% confidence intervals (CIs) for AUC_0–t_ and C_max_ would be no wider than ±2.86 h·ng/mL and ± 0.18 h·ng/mL, respectively, was eight patients.

Parts 2 and 3 of this study: a minimum sample size of 72 enrolled patients was chosen based on practical considerations and upon agreement with the Paediatric Committee of the European Medicines Agency to ensure a final sample size of at least 50 patients. This assumed that 70% of patients would be eligible based on the inclusion and exclusion criteria, which would have enabled a crossover non-inferiority comparison between LC and CC. This sample size had 81% power to show that the lower limit of the 95% CI for the difference in the percentage of patients achieving serum phosphorus targets between LC- and CC-treated patients was above the non-inferiority margin of − 18%. However, owing to difficulties with recruiting patients, the sample size was adjusted to 32 patients who completed 8 weeks of LC treatment. This led to the removal of the crossover non-inferiority comparison between LC and CC that was originally proposed in the study design.

Investigation into the safety profiles of LC and CC required a sample size of 32 patients to observe at least one event with a true event rate of 5% at a probability of 80.6%.

#### Summary statistics

Baseline and post-treatment characteristics; serum phosphorus, calcium and calcium-phosphorus product levels; LC plasma concentrations; PK parameters; and biochemical bone markers were descriptively summarized. The descriptive statistics used were number (%), mean (SD), mean (standard error of the mean [SEM]), geometric mean (percentage coefficient of variation) and median (min, max).

The percentages of patients achieving age-specific KDOQI serum phosphorus target levels after 8 weeks of treatment with LC during part 2 were descriptively summarized, and the two-sided 95% CIs were calculated using the Clopper–Pearson method.

For the secondary efficacy endpoints, data were summarized using descriptive statistics. Continuous data were reported by the number of observations, mean (SD or SEM), median (min, max) and two-sided 95% CI of the mean. Categorical data were described by the number of observations and percentages. LC plasma concentration–time data were determined by non-compartmental analyses and based on actual sampling time. Analyses were conducted using Phoenix WinNonlin Version 6.3 (Pharsight Corporation) or higher.

### Study populations

Full analysis set: patients who received at least one dose of study drug, and who had serum phosphorus data available for analysis during part 2 and/or part 3.

Per-protocol set 1: patients who received CC for 8 weeks during part 2, followed by a washout period and then LC for 8 weeks during part 2, and who had serum phosphorus data available for analysis. Only patients who had serum phosphorus levels above the age-specific KDOQI targets at study entry and between the CC and LC treatment regimens or the visits during the washout, before either part 1 or part 2, were included in this set.

Per-protocol set 2: patients who received LC for at least 8 weeks during part 2 and/or part 3, and who had serum phosphorus data available for analysis. Only patients who had serum phosphorus levels above the age-specific KDOQI targets before the start of LC treatment were included in this set.

Safety analysis set 1: patients who received one dose of LC in part 1 of the study and who attended at least one safety follow-up visit.

Safety analysis set 2: patients who received at least one dose of CC or LC in part 2 and/or part 3 of the study and who attended at least one safety follow-up visit.

Safety completer set: patients who received LC for at least 8 weeks in part 2 and/or part 3 of the study.

Pharmacokinetic set 1: patients from safety analysis set 1 who had at least one measurable plasma concentration of LC post-dose.

## Results

### Patient demographics and baseline characteristics

**Fig. 2:**
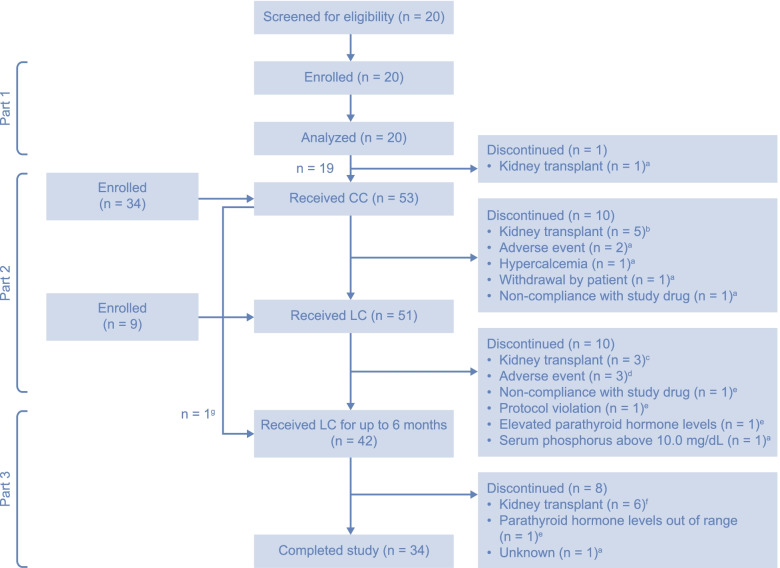
CONSORT flow diagram. ^a ^Patient was receiving hemodialysis. ^b ^Patients were receiving hemodialysis (*n* = 3) and peritoneal dialysis (*n* = 2). ^c ^Patients were receiving hemodialysis (*n* = 1) and peritoneal dialysis (*n* = 2). ^d ^Patients were receiving hemodialysis (*n* = 2) and peritoneal dialysis (*n* = 1). ^e ^Patient was receiving peritoneal dialysis. ^f ^Patients were receiving hemodialysis (*n* = 2) and peritoneal dialysis (*n* = 4). ^g ^One patient who received CC during part 2 proceeded to part 3 to receive LC for up to 6 months *CC* calcium carbonate, *CONSORT* Consolidated Standards of Reporting Trials, *LC* lanthanum carbonate

This study was conducted at 22 sites: 14 in the European Union, three in the Russian Federation, three in Latin America, one in Turkey and one in South Africa (Additional Table 1). Patient demographics and baseline characteristics are presented in Table 1. Overall, 63 patients were enrolled in this study; a summary of patient disposition is presented in Fig. 2. In part 1 (*n* = 20), the mean age (SD) was 13.1 (2.7) years; the majority of patients were white (95.0%) and half were male (50.0%). In part 2, for patients in the CC group (*n* = 53), the mean age (SD) was 13.1 (2.8) years; the majority of patients were white (98.1%) and more than half were male (54.7%). In part 2, for patients in the LC group (*n* = 51), the mean age (SD) was 13.6 (2.7) years; the majority of patients were white (98.0%) and more than half were male (58.8%). For patients treated with LC for at least 8 weeks during part 2 and/or part 3 (*n* = 46), the mean age (SD) was 13.8 (2.6) years; the majority of patients were white (97.8%) and more than half were male (58.7%). More patients were receiving hemodialysis than peritoneal dialysis (part 1, 60.0% vs 40.0%; part 2 [CC], 56.6% vs 43.4%; part 2 [LC], 56.9% vs 43.1%; parts 2 and 3 combined, 60.9% vs 39.1%) (Table 1). During part 2 of the study, 90.6 and 96.1% of CC- and LC-treated patients achieved 60–120% compliance, respectively. For parts 2 and 3 of the study combined, 96.2% of LC-treated patients achieved 60–120% compliance. Patient demographics and characteristics at baseline, stratified by dialysis mode, are reported in Additional Table 2.

**Table 1:** Baseline demographics and characteristics of CC- and LC-treated patients

Characteristic	Part 1 (Safety analysis set 1)	Part 2 (Safety analysis set 2)		Parts 2 and 3 combined (Safety completer set)
	LC (***n*** = 20)	CC (***n*** = 53)	LC (***n*** = 51)	LC (***n*** = 46)
Age, years, mean (SD)	13.1 (2.7)	13.1 (2.8)	13.6 (2.7)	13.8 (2.6)
Age group, years, mean (SD)^a^				
< 10^b^	1 (5.0)	3 (5.7)	2 (3.9)	2 (4.3)
10 to 11	4 (20.0)	11 (20.8)	9 (17.6)	6 (13.0)
12 to 17	15 (75.0)	39 (73.6)	40 (78.4)	38 (82.6)
Sex, n (%)				
Male	10 (50.0)	29 (54.7)	30 (58.8)	27 (58.7)
Female	10 (50.0)	24 (45.3)	21 (41.2)	19 (41.3)
Ethnicity, n (%)				
Hispanic or Latino	0 (0.0)	5 (9.4)	4 (7.8)	4 (8.7)
Non-Hispanic or Latino	20 (100)	48 (90.6)	47 (92.2)	42 (91.3)
Race, n (%)				
White	19 (95.0)	52 (98.1)	50 (98.0)	45 (97.8)
Non-white	1 (5.0)	1 (1.9)	1 (2.0)	1 (2.2)
Weight, kg, mean (SD)	41.9 (16.5)	38.7 (15.1)	41.7 (15.9)	42.4 (16.1)
Height, cm, mean (SD)	149.2 (19.3)	145.7 (19.7)	149.1 (19.0)	149.9 (19.1)
Body mass index, kg/m^2^, mean (SD)	18.1 (3.4)	17.7 (3.2)	18.3 (3.9)	18.4 (4.0)
Dialysis mode, n (%)				
Hemodialysis	12 (60.0)	30 (56.6)	29 (56.9)	28 (60.9)
Peritoneal dialysis	8 (40.0)	23 (43.4)	22 (43.1)	18 (39.1)

Safety analysis set 1 included all patients who received at least one dose of LC in part 1 and attended at least one safety follow-up visit. Safety analysis set 2 included all patients who received at least one dose of CC or LC in part 2 and/or part 3 of the study and attended at least one safety follow-up visit. The safety completer set included all patients who received LC for at least 8 weeks in part 2 and/or part 3 of the study

^a ^Age group was calculated as the difference between the date of birth and the date that informed consent/assent was received

^b ^Patient demographics are presented for patients < 10 years old who were included in the study before the protocol amendment, in which the inclusion criteria of 6 months to < 18 years old was adjusted to 10 years to < 18 years old

*CC* calcium carbonate, *LC* lanthanum carbonate, *SD* standard deviation

### Age-specific KDOQI serum phosphorus target levels

**Fig. 3:**
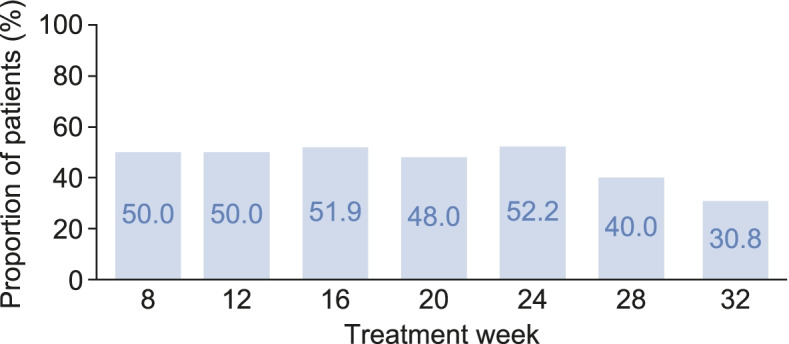
Proportion of LC-treated patients achieving age-specific KDOQI serum phosphorus target levels by treatment week. Age-specific KDOQI serum phosphorus target levels were defined as: ≤1.78 mmol/dL for adolescents ≥12 years to < 18 years old and ≤ 1.94 mmol/dL for children ≥10 years to < 12 years old. Data are presented for patients from per-protocol set 2, which included all patients who received LC for at least 8 weeks during part 2 and/or part 3, and who had serum phosphorus data available for analysis, and calculated based on the number of patients with serum phosphorus data available for each time point *LC* lanthanum carbonate, *KDOQI* Kidney Disease Outcomes Quality Initiative

For the primary efficacy endpoint, after 8 weeks of LC treatment during part 2 and/or part 3 of the study, 18 patients (34.6%) from the full analysis set and 17 patients (50.0%) from per-protocol set 1 met KDOQI serum phosphorus target levels (Table 2). After 8 weeks of CC treatment (during part 2), 10 patients (58.8%) from per-protocol set 1 met KDOQI serum phosphorus target levels; after a further 8 weeks of LC treatment (during part 2 and after a washout period of up to 3 weeks), 12 patients (70.6%) met KDOQI serum phosphorus target levels (Table 2). Overall, there was a decrease in the proportion of patients from per-protocol set 2 achieving age-specific KDOQI serum phosphorus target levels from week 8 to week 32 of LC treatment (during parts 2 and/or 3) (Fig. 3). For patients who received at least 8 weeks of LC (per-protocol set 2), the proportion of patients achieving serum phosphorus control was in the range of 30–50% during up to 32 weeks of treatment with LC for patients with available serum phosphorus data. The proportion of patients who received 8 weeks of CC followed by 8 weeks of LC and who did not have serum phosphorus data available or did not satisfy other eligibility criteria at week 8, are presented in Additional Fig. 1.

**Table 2:** Proportion of patients achieving age-specific KDOQI serum phosphorus target levels after 8 weeks of treatment

	Per-protocol set 1		Per-protocol set 2	Full analysis set^**c**^
	CC (***n*** = 17)	LC (***n*** = 17)	LC (***n*** = 34)	LC (***n*** = 52)
Responders, n (%)^a^	10 (58.8)	12 (70.6)	17 (50.0)	18 (34.6)
95% CI for percentage of responders^b^	32.9, 81.6	44.0, 89.7	32.4, 67.6	22.0, 49.1

Recorded 8-week treatment periods occurred during part 2 and/or part 3 of the study. Per-protocol set 1 included all patients who received CC for 8 weeks during part 2, followed by a washout period and then LC for at least 8 weeks during part 2, and who had serum phosphorus data available for analysis. Per-protocol set 2 included all patients who received LC for at least 8 weeks during part 2 and/or part 3, and who had serum phosphorus data available for analysis. The full analysis set included all patients who received at least one dose of study drug during part 2 and/or part 3 and who had serum phosphorus data available for analysis. The primary efficacy endpoint is shaded in grey

^a ^Responders were defined as patients with KDOQI serum phosphorus levels of ≤1.78 mmol/L (patients ≥12 years to < 18 years old) and ≤ 1.94 mmol/L (patients ≥10 years to < 12 years old)

^b ^The 95% CI for the percentage of responders was calculated using the Clopper–Pearson method

^c ^For patients who had missing central laboratory data, local laboratory data were used, if available; otherwise, patients were excluded from relevant analyses

*CC* calcium carbonate, *CI* confidence interval, *KDOQI* Kidney Disease Outcomes Quality Initiative, *LC* lanthanum carbonate

### Serum phosphorus, calcium and calcium-phosphorus product levels

Changes in serum phosphorus, calcium and calcium-phosphorus product levels were monitored for patients from per-protocol set 1. Mean (SEM) changes after 8 weeks of CC were generally similar to those after 8 weeks of LC; however, changes in calcium-phosphorus product levels were greater for CC-treated patients (Table 3).

**Table 3:** Changes from baseline in serum phosphorus, calcium and calcium-phosphorus product levels

Treatment arm	Phosphorus, mmol/L	Calcium, mmol/L	Calcium-phosphorus, mmol^**2**^/L^**2**^
**Per-protocol set 1 – change from baseline to week 8**^**a**^			
CC (*n* = 17)			
Baseline	2.165 (0.093)	2.319 (0.052)	4.904 (0.247)
Week 8^b^	1.633 (0.141)	2.384 (0.077)	3.917 (0.412)
Change from baseline^c^	−0.520 (0.179)	0.058 (0.055)	−0.966 (0.408)
LC (*n* = 17)			
Baseline	2.188 (0.087)	2.371 (0.065)	4.729 (0.350)
Week 8	1.721 (0.107)	2.362 (0.052)	4.060 (0.256)
Change from baseline	−0.467 (0.131)	−0.009 (0.057)	−0.669 (0.383)
**Per-protocol set 2 – change from baseline by week**^**c**^			
LC (*n* = 34; n varies by week and measurement)			
Baseline	2.274 (0.072)	2.426 (0.041)	5.275 (0.231)
Week 8	1.940 (0.098)	2.424 (0.037)	4.698 (0.236)
Change from baseline	−0.334 (0.104)	− 0.001 (0.036)	− 0.577 (0.246)
Week 12^d^	1.833 (0.076)	2.359 (0.043)	4.341 (0.210)
Change from baseline^d^	−0.416 (0.091)	−0.045 (0.048)	−0.783 (0.254)
Week 16^e^	1.941 (0.114)	2.359 (0.060)	4.580 (0.277)
Change from baseline^e^	−0.314 (0.128)	−0.034 (0.053)	−0.575 (0.302)
Week 20^f^	1.898 (0.085)	2.397 (0.055)	4.660 (0.287)
Change from baseline^f^	−0.316 (0.106)	0.003 (0.051)	−0.362 (0.271)
Week 24^g^	1.884 (0.138)	2.371 (0.051)	4.521 (0.349)
Change from baseline^g^	−0.322 (0.136)	−0.010 (0.045)	−0.578 (0.346)
Week 28^h^	1.953 (0.125)	2.381 (0.074)	4.658 (0.331)
Change from baseline^h^	−0.236 (0.149)	0.049 (0.062)	−0.326 (0.377)
Week 32^i^	2.008 (0.142)	2.210 (0.061)	4.484 (0.345)
Change from baseline^i^	−0.143 (0.156)	−0.127 (0.077)	−0.570 (0.362)

All data are presented as mean (SEM). Per-protocol set 1 included all patients who received CC for 8 weeks during part 2, followed by a washout period and then LC for at least 8 weeks during part 2, and who had serum phosphorus data available for analysis. Per-protocol set 2 included all patients who received LC for at least 8 weeks during part 2 and/or part 3, and who had serum phosphorus data available for analysis. Patients with missing data were excluded from these analyses

^a ^One patient did not complete the 8 weeks of CC treatment and progressed straight to the 8 weeks of LC treatment

^b ^*n* = 16

^c ^Weeks listed are exposure to LC and not the scheduled visit

^d ^Phosphorus, *n* = 30; calcium, *n* = 28; calcium-phosphorus product, *n* = 28

^e ^Phosphorus, *n* = 27; calcium, *n* = 26; calcium-phosphorus product, *n* = 26

^f ^Phosphorus, *n* = 25; calcium, *n* = 22; calcium-phosphorus product, *n* = 22

^g ^Phosphorus, *n* = 23; calcium, *n* = 22; calcium-phosphorus product, *n* = 22

^h ^*n* = 20

^i ^*n* = 13

*CC* calcium carbonate, *LC* lanthanum carbonate, *SEM* standard error of the mean

For patients from per-protocol set 2 who received LC for at least 8 weeks during part 2 and/or part 3 of the study (*n* = 34), mean (SEM) changes from baseline to week 8 were: phosphorus, − 0.334 (0.104) mmol/L; calcium, − 0.001 (0.036) mmol/L; and calcium-phosphorus, − 0.577 (0.246) mmol^2^/L^2^ (Table 3). Over the 6-month extension treatment period, mean (SEM) serum phosphorus and calcium-phosphorus product levels were maintained below baseline up to week 32 (mean [SEM] changes from baseline to week 12: phosphorus, − 0.416 [0.091]; calcium-phosphorus, − 0.783 [0.254]; week 16: phosphorus, − 0.314 [0.128]; calcium-phosphorus, − 0.575 [0.302]; week 20: phosphorus, − 0.316 [0.106]; calcium-phosphorus, − 0.362 [0.271]; week 24: phosphorus, − 0.322 [0.136]; calcium-phosphorus, − 0.578 [0.346]; week 28: phosphorus, − 0.236 [0.149]; calcium-phosphorus, − 0.326 [0.377]; week 32: phosphorus, − 0.143 [0.156]; calcium-phosphorus, − 0.570 [0.362]). In general, there was no change in serum calcium levels over the 6-month extension period (Table 3). The proportion of patients who received at least 8 weeks of LC and who did not have serum phosphorus data available or did not satisfy other eligibility criteria at weeks 8, 12, 16, 20, 24, 28 and 32, are presented in Additional Fig. 1.

### Pharmacokinetic analysis

LC plasma concentration data were available for all patients from pharmacokinetic set 1 during part 1 (*n* = 20). Data were analyzed from before administration to up to 48 h after a single oral dose of LC (500 mg, patients ≤12 years old; 1000 mg, patients > 12 years old).

**Fig. 4:**
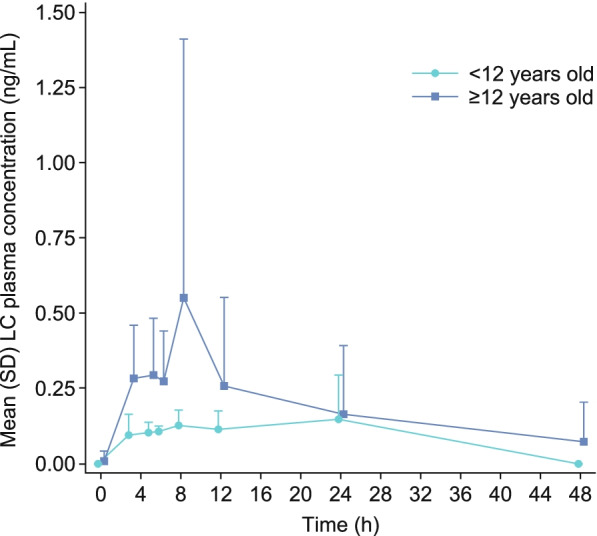
LC plasma concentrations for patients after a single oral dose (part 1, *n* = 20). Pharmacokinetic set 1 included all patients from safety analysis set 1 who had at least one measurable plasma concentration of LC post-dose. Patients with LC plasma concentrations below the lower limit of quantification (0.05 ng/mL) were not included at the respective time points; lower SD error bars have been omitted for clarity *LC* lanthanum carbonate, *SD* standard deviation

LC was absorbed slowly, with t_max_ generally occurring within 3–8 h; however, t_max_ was measured as late as 12–24 h in some patients (Table 4 and Fig. 4). The mean t_1/2_ of LC could not be calculated for all patients owing to missing data (4 patients younger than 12 years old had missing data); however, on average, t_1/2_ (percentage of covariance) varied substantially between patients 12 years and older, and occurred later in patients 12 years and older (17.07 [66.91] h) than in the patient younger than 12 years old (8.83 [N/A] h) (Table 4). The single-dose PK profile of LC in pediatric patients was highly variable between individuals and had large percentages of covariance.

**Table 4:** Pharmacokinetic parameters after a single oral dose of LC

Parameter	Age groups	
	< 12 years^**a**^ (***n*** = 5)	12–17 years^**a**^ (***n*** = 15)
AUC_0–inf_ (h·ng/mL)		
n	1	10
Geometric mean (CV%)	1.93 (N/A)	6.64 (51.41)
AUC_0–48_ (h·ng/mL)		
n	2	12
Geometric mean (CV%)	1.96 (39.73)	6.03 (110.88)
AUC_last_ (h·ng/mL)		
n	5	15
Geometric mean (CV%)	2.36 (40.16)	5.59 (109.48)
C_max_ (ng/mL)		
n	5	15
Geometric mean (CV%)	0.20 (43.59)	0.43 (131.56)
t_max_ (h)		
n	5	15
Median (min, max)	8.00 (3.00, 23.87)	5.00 (2.97, 12.00)
t_1/2_ (h)		
n	1	10
Geometric mean (CV%)	8.83 (N/A)	17.07 (66.91)
CL/F (L/h)		
N	1	10
Geometric mean (CV%)	258,644.20 (N/A)	150,545.92 (95.06)
V_z_/F (L)		
N	1	10
Geometric mean (CV%)	3,295,611.00 (N/A)	3,706,259.60 (51.46)
λ_z_		
N	1	10
Geometric mean (CV%)	0.08	0.04

Data are presented for patients from pharmacokinetic set 1, which included all patients from safety analysis set 1 who had at least one measurable plasma concentration of LC post-dose who received a single oral dose of LC during part 1 of the study, stratified by age. For patients who had missing central laboratory data, local laboratory data were used, if available; otherwise, patients were excluded from relevant analyses

^a ^Patients aged < 12 years and patients 12–17 years received a single dose of 500 mg and 1000 mg of LC, respectively

*λ*_*z*_ first-order rate constant associated with the terminal (log-linear) portion of the curve, *AUC*_*0–48*_ area under the curve from the time of dosing to 48 h after dosing, *AUC*_*0–inf*_ area under the curve extrapolated to infinity, calculated using the last measurable concentration, *AUC*_*last*_ area under the curve from the time of dosing to the last measurable concentration, *CL/F* total body clearance for extravascular administration divided by the fraction of dose absorbed, *C*_*max*_ maximum observable plasma concentration, *CV%* percentage coefficient of variation, *LC* lanthanum carbonate, *N/A* not available, *t*_*1/2*_ terminal half-life; *t*_*max*_ time of maximum observed plasma concentration, *V*_*z*_*/F* volume of distribution associated with the terminal slope after extravascular administration divided by the fraction of dose absorbed

### Safety assessments

During part 2 of the study, mean (SD) total daily doses of CC and LC were 2128.7 (1061.6) mg and 1629.6 (504.6) mg, respectively. The mean (SD) lengths of exposure were 50.6 (17.7) days and 68.9 (23.3) days, respectively. In parts 2 and 3 of the study combined, the mean (SD) total daily dose of LC and length of exposure were 1704.8 (604.1) mg and 187.0 (90.2) days, respectively.

During part 1 of the study, treatment-emergent adverse events (TEAEs) were reported in 10.0% (*n* = 2/20) of patients who received a single oral dose of LC; none of these were reported as serious. The most common non-serious TEAEs during part 1 were nausea (5.0% [*n* = 1/20]), vomiting (5.0% [*n* = 1/20]) and somnolence (5.0% [*n* = 1/20]), all cases of which were considered mild (data not shown).

A summary of the overall TEAEs is presented in Table 5 for parts 2 and 3 of the study. During part 2, reports of TEAEs over 8 weeks of treatment were similar between CC- and LC-treated patients (52.8% [*n* = 28/53] vs 54.9% [*n* = 28/51]). Overall, for patients receiving LC during part 2 and/or part 3 of the study, 78.8% (*n* = 41/52) of patients experienced a TEAE.

**Table 5:** Frequently reported TEAEs (≥3% of patients in ≥1 treatment arm) in parts 2 and 3

Category of TEAE	Safety analysis set 2 during part 2 of the study^**a**^				Safety analysis set 2 during part 2 and/or part 3 of the study^**a**^	
	CC (***n*** = 53)		LC (***n*** = 51)		LC (***n*** = 52)	
	n (%)	m	n (%)	m	n (%)	m
Any	28 (52.8)	63	28 (54.9)	56	41 (78.8)	124
Serious	9 (17.0)	10	11 (21.6)	17	19 (36.5)	29
Related to study drug	10 (18.9)	16	8 (15.7)	14	12 (23.1)	21
Leading to study withdrawal	2 (3.8)	2	3 (5.9)	6	3 (5.8)	6
Leading to death	0 (0.0)	0	0 (0.0)	0	0 (0.0)	0
TEAEs occurring in ≥3% of patients in ≥1 treatment arm						
Blood and lymphatic system disorders						
Anemia	0 (0.0)	0	0 (0.0)	0	2 (3.8)	2
Gastrointestinal disorders						
Vomiting^b^	1 (1.9)	2	2 (3.9)	2	6 (11.5)	7
Nausea^c^	2 (3.8)	2	2 (3.9)	3	4 (7.7)	6
Abdominal pain	1 (1.9)	1	0 (0.0)	0	2 (3.8)	3
Diarrhea	2 (3.8)	3	0 (0.0)	0	0 (0.0)	0
General disorders and administration site conditions						
Pyrexia	0 (0.0)	0	1 (2.0)	1	2 (3.8)	3
Infections and infestations						
Peritonitis^d^	0 (0.0)	0	3 (5.9)	4	5 (9.6)	6
Upper respiratory tract infection	0 (0.0)	0	3 (5.9)	3	5 (9.6)	5
Nasopharyngitis	2 (3.8)	2	0 (0.0)	0	2 (3.8)	3
Device related infection	0 (0.0)	0	2 (3.9)	2	2 (3.8)	3
Respiratory tract infection	3 (5.7)	5	0 (0.0)	0	2 (3.8)	2
Hordeolum	0 (0.0)	0	1 (2.0)	1	2 (3.8)	2
Influenza	2 (3.8)	2	0 (0.0)	0	1 (1.9)	1
Metabolism and nutrition disorders						
Hypercalcemia^e^	9 (17.0)	9	4 (7.8)	4	5 (9.6)	9
Hypocalcemia	3 (5.7)	3	1 (2.0)	1	3 (5.8)	6
Hyperkalemia^e^	1 (1.9)	1	0 (0.0)	0	2 (3.8)	2
Hyperphosphatemia	2 (3.8)	2	0 (0.0)	0	0 (0.0)	0
Hypophosphatemia	3 (5.7)	4	4 (7.8)	5	6 (11.5)	10
Vascular disorders						
Hypotension	0 (0.0)	0	2 (3.9)	4	2 (3.8)	4
Hypertension^d^	3 (5.7)	3	1 (2.0)	1	1 (1.9)	1

Safety analysis set 2 included all patients who received at least one dose of CC or LC in part 2 and/or part 3 of the study and attended at least one follow-up visit. Reported TEAEs are stratified by treatment group and by system organ class and preferred term. Most TEAEs were considered by the investigator to be mild or moderate in severity, unless otherwise indicated

^a ^TEAEs were categorized by the treatment most recently received by the patient and patients were counted once per category, per treatment group; adverse events were considered TEAEs if they occurred in the 3 weeks after the most recent dose of study drug within the relevant part of the study

^b ^TEAEs were classified as severe in one LC-treated patient (2.0%) in part 2, and one LC-treated patient (1.9%) in parts 2 and 3 combined

^c ^TEAEs were classified as severe in one LC-treated patient (2.0%) in part 2, and one LC-treated patient (1.9%) in parts 2 and 3 combined

^d ^TEAEs were classified as severe in two LC-treated patients (3.9%) in part 2, and three LC-treated patients (5.8%) in parts 2 and 3 combined

^e ^TEAEs were classified as severe in one CC-treated patient (1.9%) in part 2

*CC* calcium carbonate, *LC* lanthanum carbonate, *m* the number of events experienced, *TEAE* treatment-emergent adverse event

The majority of TEAEs reported throughout the study were of mild or moderate severity; no deaths or TEAEs of special interest were reported (Table 5). During part 2 of the study, serious TEAEs were reported in nine patients (17.0%) and 11 patients (21.6%) in the CC and LC treatment groups, respectively. The most common serious TEAE was hypertension (3.8% [*n* = 2/53]) for CC-treated patients and peritonitis (5.9% [*n* = 3/51]) for LC-treated patients during part 2. For LC-treated patients in part 2 and/or 3 of the study, 19 patients (36.5%) reported serious TEAEs; the most common serious TEAE was also peritonitis (9.6% [*n* = 5/52]).

During part 2, the most commonly experienced non-serious TEAEs for CC-treated patients were hypercalcemia (17.0% [*n* = 9/53]), respiratory tract infection (5.7% [*n* = 3/53]), hypocalcemia (5.7% [*n* = 3/53]), hypophosphatemia (5.7% [*n* = 3/53]) and hypertension (5.7% [*n* = 3/53]). However, for LC-treated patients in part 2, these were hypercalcemia (7.8% [*n* = 4/51]), hypophosphatemia (7.8% [*n* = 4/51]), peritonitis (5.9% [*n* = 3/51]) and upper respiratory tract infection (5.9% [*n* = 3/51]). For LC-treated patients in part 2 and/or part 3, the most common non-serious TEAEs were vomiting (11.5% [*n* = 6/52]), hypophosphatemia (11.5% [*n* = 6/52]), peritonitis (9.6% [*n* = 5/52]), upper respiratory tract infection (9.6% [*n* = 5/52]), hypercalcemia (9.6% [*n* = 5/52]), nausea (7.7% [*n* = 4/52]) and hypocalcemia (5.8% [*n* = 3/52]).

There were relatively few TEAEs that led to study withdrawal (Table 5). During part 2 of the study, two CC-treated patients withdrew owing to TEAEs of acute hepatitis and hypercalcemia (1.9% [*n* = 1/53] each). During part 2 and/or part 3 of the study, three LC-treated patients withdrew owing to TEAEs of: peritonitis (2 events) and perforated appendicitis (1 event) in the first patient; lip edema (1 event) and skin rash (1 event) in the second patient; and vomiting (1 event) in the third patient. All events were considered by the investigator to be related to the study drug; peritonitis was the only TEAE to occur twice in the same patient.

The number of TEAEs considered by the investigators to be related to the study drug was low (see Additional Table 3). One LC-treated patient experienced a serious adverse event of peritonitis during part 2 of the study that was considered related to LC.

No statistically significant differences were observed between CC- and LC-treated patients in change in height and weight (data not shown) or biochemical bone markers over 8 weeks of treatment (see Additional Table 4), and no bone disease-related TEAEs were reported in any part of the study. Overall, CC and LC were generally well tolerated. Reports of adverse events were generally similar between patients who were receiving hemodialysis compared with patients who were receiving peritoneal dialysis (Additional Table 5). Peritonitis and upper respiratory tract infection occurred predominantly in patients who were receiving peritoneal dialysis, and metabolism and nutrition disorders, such as hypercalcemia, hypocalcemia and hypophosphatemia, were more common in patients who were receiving hemodialysis compared with patients who were receiving peritoneal dialysis (Additional Table 5).

## Discussion

This is the first study to assess the PK of a single oral dose of LC in a pediatric population. It is also the first study to address the effect of LC on serum phosphorus, calcium and calcium-phosphorus product levels in pediatric patients with advanced CKD undergoing dialysis. For the primary efficacy analysis, 50.0% of patients met age-specific KDOQI serum phosphorus target levels after 8 weeks of treatment with LC during part 2 and/or part 3 of the study. For the secondary efficacy endpoints, the proportions of patients who met age-specific KDOQI serum phosphorus target levels were 58.8% after 8 weeks of treatment with CC, and 70.6% after a subsequent washout and a further 8 weeks of treatment with LC. Changes in serum phosphorus and calcium levels were similar for CC- and LC-treated patients after 8 weeks of therapy. Although decreases in calcium-phosphorus product levels were greater for CC- than LC-treated patients, calcium-phosphorus product levels did not return to baseline levels during the 4-week washout, and serum levels at week 8 were similar for CC- and LC-treated patients. LC was absorbed slowly with t_max_ occurring between 3 and 24 h; the single-dose PK profile of LC was highly variable between individuals in this cohort. Finally, CC and LC were well-tolerated; the majority of TEAEs were mild to moderate in severity and did not result in study drug withdrawal or study discontinuation. Together, these data support the efficacy and tolerability of LC as a phosphate binder for use in the treatment of pediatric patients with hyperphosphatemia and CKD undergoing dialysis.

The efficacy of LC for the treatment of hyperphosphatemia in pediatric patients was found to be consistent with a previous study with equivalent dosing of LC in adults with CKD [32] and pediatric patients treated with CC [28]. The majority of patients who received 8 weeks of CC followed by 8 weeks of LC in part 2 met KDOQI serum phosphorus target levels after 8 weeks of CC (58.8%) and after 8 weeks of LC (70.6%). Serum phosphorus control was generally maintained over the 6-month extension period. A previous study of LC (chewable tablet formulation) in adults with CKD showed that a higher percentage of LC-treated patients achieved KDOQI serum phosphorus target levels versus placebo (44.6% vs 26.5%) [32]; these results are consistent with the findings from patients from per-protocol set 2 of this study, in which 50.0% of patients receiving LC achieved serum phosphorus control after 8 weeks.

Clinically meaningful reductions in serum phosphorus and calcium-phosphorus product levels from baseline to week 8 of treatment were measured in LC-treated patients during part 2. These results are similar to those reported previously for LC-treated adult patients, in whom the mean (SD) change from baseline in serum phosphorus concentrations was − 0.18 (0.03) mmol/L [32]. The mean reduction in serum calcium-phosphorus product levels was greater for CC-treated patients during part 2 than for LC-treated patients in part 2 and/or part 3. Elevated serum calcium-phosphorus product levels have been linked to an increased risk of death of 40–50% in patients on peritoneal dialysis and hemodialysis [33]. As expected, serum calcium levels were maintained in LC-treated pediatric patients during part 2 of the study. This finding is consistent with serum calcium levels reported for LC-treated adults in a previous study [32], and is reflective of the lack of reported adverse events for LC with respect to calcium metabolism.

After oral administration, LC was absorbed slowly, with t_max_ typically occurring within 3–8 h, but as late as 12–24 h in some patients; the geometric mean t_1/2_ was approximately 17 h after dosing in patients greater than 12 years old. There was high PK variability in the pediatric population, particularly for C_max_ and AUC, which had coefficients of variation greater than 100%.

The majority of TEAEs were mild to moderate in severity, and the proportion of patients who had TEAEs considered by the investigator to be related to the study drug was low. Relatively few adverse events led to study withdrawal and no deaths occurred during the study. In the current study, peritonitis was only reported as a TEAE in patients who were receiving LC. The safety profile of LC is also similar to that of sucroferric oxyhydroxide (SFOH) and calcium acetate (CaAc), as reported in a recent study of pediatric patients with CKD for up to 34 weeks [34]. During this study, 75.8 and 73.7% of SFOH- and CaAc-treated patients, respectively, experienced at least one TEAE, [34] compared with 78.8% of LC-treated patients in our study. Furthermore, the proportions of patients who discontinued the study owing to TEAEs were higher for SFOH and CaAc (18.2 and 31.6%, respectively) [34], than for LC (5.8%).

Biochemical bone markers were monitored because links have been indicated between bone metabolism and cardiovascular calcification, morbidity and mortality in patients with CKD [29, 30]. A previous clinical study demonstrated that treatment with LC has the potential to prevent low bone turnover in comparison with treatment with CC [35], as shown by changes in parathyroid hormone levels (see Additional Table 4). Furthermore, the potential for heavy metal accumulation within bones, such as aluminum and lanthanum, in children with growing skeletons could be of concern to healthcare providers [25]. However, during this study, the mean changes from baseline in height, weight and biochemical bone markers were consistent between CC- and LC-treated groups over 8 weeks of treatment. A 10-year safety analysis also demonstrated that LC is unlikely to show aluminum-like accumulation and there is currently no evidence of an association between LC and bone toxicity [22]. This 10-year safety analysis also demonstrated that plasma lanthanum levels are not statistically different for LC doses of 750–3000 mg, indicating a non-linear relationship between the doses of LC used in our study and LC plasma concentrations [22].

The strengths of this trial are that it comprehensively summarized the efficacy, safety and PK profile of LC in pediatric patients who were treated over a long period of time. Furthermore, this pediatric study is a continuation of a robust program of 45 completed clinical trials of LC (20 clinical pharmacology studies, four placebo-controlled phase 2 studies, four phase 3 studies, one long-term safety study, six safety extension studies, four phase 3b studies, four phase 4 studies and two post-marketing surveillance studies). In completed phase 2–4 studies, 5533 patients with CKD undergoing dialysis have been treated with multiple doses of LC.

A limitation of this study is that, owing to recruitment difficulties, there was an insufficient number of patients to permit direct comparisons between CC and LC treatment arms. Recruitment was challenging due to the limited population of pediatric patients who met the eligibility criteria globally. In addition, the proportion of patients who were missing serum phosphorus data, or did not satisfy other eligibility criteria, increased during part 3, and assignment of the study drugs was not randomized.

## Conclusions

In summary, half of the pediatric patients with CKD undergoing dialysis achieved therapeutic control of serum phosphorus levels, as defined by the KDOQI guidelines, after 8 weeks of treatment with LC during part 2. These findings were consistent with data from adult patients with CKD in previous studies. Parts 2 and 3 of this study showed that serum phosphorus levels were maintained below baseline for up to 32 weeks. LC (oral powder formulation) was generally well tolerated, and no new safety signals were identified in pediatric patients with CKD; these findings were consistent with those from adults with CKD in other studies of LC. These data suggest that LC is an effective phosphate binder for the treatment of hyperphosphatemia in pediatric patients with CKD undergoing dialysis.

## Supplementary Information

**Additional file 1: Figure 1.** Proportion of patients who did not have serum phosphorus data available or who did not satisfy other eligibility criteria^a^ after 8 weeks of CC followed by 8 weeks of LC (A) and after at least 8 weeks of LC (B); these patients were thus excluded from the efficacy analyses (per-protocol sets 1 and 2).

**Additional file 2: Table 1.** List of center that participated in this study.

**Additional file 3: Table 1.** Baseline demographics and characteristics of CC- and LC-treated patients, stratified by dialysis mode.

**Additional file 4: Table 1.** TEAEs considered related to study drug, experienced by ≥1 patient in ≥1 treatment arm.

**Additional file 5: Table 1.** Change from baseline in biochemical bone markers for patients in part 2 of the study^a^.

**Additional file 6: Table 1.** Frequently reported TEAEs (≥10% of patients in ≥1 treatment arm) in parts 2 and 3 of the study, stratified by dialysis modality.

## Data Availability

The datasets generated and analyzed during the current study are available in the ClinicalTrials.gov repository, https://clinicaltrials.gov/ct2/show/results/NCT01696279.

## Abbreviations

AUC: Area under the curve
CaAc: Calcium acetate
CC: Calcium carbonate
CI: Confidence interval
CKD: Chronic kidney disease
ESRD: End-stage renal disease
KDOQI: Kidney Disease Outcomes Quality Initiative
LC: Lanthanum carbonate
PK: Pharmacokinetics
q.d.: Once daily
SD: Standard deviation
SEM: Standard error of the mean
SFOH: Sucroferric oxyhydroxide
TEAE: Treatment-emergent adverse event
