# Supplementary material for: An open-label phase 2 trial to assess the efficacy, safety and pharmacokinetics of lanthanum carbonate in hyperphosphatemic children and adolescents with chronic kidney disease undergoing dialysis
Source: BMC Nephrol. 2022 Mar 2;23:84. doi: 10.1186/s12882-022-02688-9 (PMC8892701; doi:10.1186/s12882-022-02688-9)
Supplement: Supplementary file 1 — Additional file 1: Figure 1. Proportion of patients who did not have serum phosphorus data available or who did not satisfy other eligibility criteriaa after 8 weeks of CC followed by 8 weeks of LC (A) and after at least 8 weeks of LC (B); these patients were thus excluded from the efficacy analyses (per-protocol sets 1 and 2). [file 12882_2022_2688_MOESM1_ESM.docx]

**Additional figure 1**

**An open-label phase 2 trial to assess the efficacy, safety and pharmacokinetics of lanthanum carbonate in hyperphosphatemic children and adolescents with chronic kidney disease undergoing dialysis**

Anna Wasilewska^1*^, RoseAnn Murray^2^, Aimee Sundberg^2^, Sharif Uddin^3^, Heinrich Achenbach^4^, Aleksey Shavkin^5^, Tamás Szabó^6^, Andrea Vergani^2^ and Obi Umeh^2^

*Correspondence: [anna.wasilewska@udsk.pl](mailto:anna.wasilewska@udsk.pl)

^1^Department of Pediatrics and Nephrology, Faculty of Medicine, Medical University of Bialystok, University Children’s Clinical Hospital of Bialystok, Waszyngtona, Bialystok, Poland
^2^Shire Human Genetic Therapies, Inc., a Takeda company, Cambridge, MA, USA
^3^Takeda Pharmaceuticals USA, Inc., Lexington, MA, USA

^4^Shire Human Genetic Therapies, Inc., a Takeda company, Zug, Switzerland ^5^Saint Petersburg State Budgetary Healthcare Institution, Children’s City Multidisciplinary Clinical Specialized Center of High Medical Technologies, Saint Petersburg, Russia
^6^Department of Pediatrics, Faculty of Medicine, University of Debrecen, Debrecen, Hungary

**Figure 1** Proportion of patients who did not have serum phosphorus data available or who did not satisfy other eligibility criteria^a^ after 8 weeks of CC followed by 8 weeks of LC (A) and after at least 8 weeks of LC (B); these patients were thus excluded from the efficacy analyses (per-protocol sets 1 and 2)

**A**  **B**

^a^Eligibility criteria included: age requirements at study entry; age-specific KDOQI targets; and/or had data available at baseline or at each timepoint assessed. Patients who did not meet these criteria were excluded from the efficacy analyses.

Per-protocol set 1 included all patients who received CC for 8 weeks during part 2, followed by a washout period and then LC for at least 8 weeks during part 2, and who had serum phosphorus data available for analysis. Only patients who had serum phosphorus levels above the age-specific KDOQI targets at study entry and between the CC and LC treatment regimens or the visits during the washout, before either part 1 or part 2, were included in this set. Per-protocol set 2 included all patients who received LC for at least 8 weeks during part 2 and/or part 3, and who had serum phosphorus data available for analysis. Only patients who had serum phosphorus levels above the age-specific KDOQI targets before the start of LC treatment were included in this set.

*CC*, calcium carbonate; *KDOQI*, Kidney Disease Outcomes Quality Initiative; *LC*, lanthanum carbonate
