# Supplementary material for: An open-label phase 2 trial to assess the efficacy, safety and pharmacokinetics of lanthanum carbonate in hyperphosphatemic children and adolescents with chronic kidney disease undergoing dialysis
Source: BMC Nephrol. 2022 Mar 2;23:84. doi: 10.1186/s12882-022-02688-9 (PMC8892701; doi:10.1186/s12882-022-02688-9)
Supplement: Supplementary file 2 — Additional file 2: Table 1. List of center that participated in this study. [file 12882_2022_2688_MOESM2_ESM.docx]

**Additional table 1**

**An open-label phase 2 trial to assess the efficacy, safety and pharmacokinetics of lanthanum carbonate in hyperphosphatemic children and adolescents with chronic kidney disease undergoing dialysis**

Anna Wasilewska^1*^, RoseAnn Murray^2^, Aimee Sundberg^2^, Sharif Uddin^3^, Heinrich Achenbach^4^, Aleksey Shavkin^5^, Tamás Szabó^6^, Andrea Vergani^2^ and Obi Umeh^2^

*Correspondence: [anna.wasilewska@udsk.pl](mailto:anna.wasilewska@udsk.pl)

^1^Department of Pediatrics and Nephrology, Faculty of Medicine, Medical University of Bialystok, University Children’s Clinical Hospital of Bialystok, Waszyngtona, Bialystok, Poland
^2^Shire Human Genetic Therapies, Inc., a Takeda company, Cambridge, MA, USA
^3^Takeda Pharmaceuticals USA, Inc., Lexington, MA, USA

^4^Shire Human Genetic Therapies, Inc., a Takeda company, Zug, Switzerland ^5^Saint Petersburg State Budgetary Healthcare Institution, Children’s City Multidisciplinary Clinical Specialized Center of High Medical Technologies, Saint Petersburg, Russia
^6^Department of Pediatrics, Faculty of Medicine, University of Debrecen, Debrecen, Hungary

**Table 1** List of centres that participated in this study

| Centro Infantil del Rinon S.r.l, Monteagudo 726, San Miguel De Tucuman 4000, Argentina |
| --- |
| Children City Clinical hospital of Saint Vladimir, 1/3, Rubtsovsko-Dvortsovay Str, Moscow 107014, Russia |
| Debreceni Egyetem Orvos- es, Egeszsegtudomanyi Centrum, Gyermekgyogyaszati Intezet, Nagyerdei Krt. 98, Debrecen 4032, Hungary |
| Dzieciecy Szpital Kliniczny im. Prof. A. Gebali,Klinika, Pediatrii I Nefrologii z, Ul. Chodzki 3, Lublin 20–093, Poland |
| Fakultni Nemocnice Ostrava, 17.listopadu 1790, Ostrava 708 52, Czech Republic |
| Gyermekgyogyaszati Klinika Es, Gyermek Egeszsegugyi Kozpont, Koranyi U. 14–15, Szeged 6720, Hungary |
| Hospital Luis Calvo Mackenna, Antonio Varas 360, Providencia, Santiago 7500539, Chile |
| Hospital Dr. Sotero del Rio, Av Concha Y Toro 3459, Puente Alto, Santiago 8207257,Chile |
| Kinder- und Jugendklinik Erlangen Studienzentrale, 2. Og, Zi. 511, Loschgestr, 15, Erlangen 91054, Germany |
| Manisa Celal Bayar University Hafsa Sultan Hospital, Uncubozköy Yerleskesi, Manisa 45030, Turkey |
| Medizinische Hochschule Hannover Klinik Für Pädiatrische Nieren-, Leber- Und Stoffwechselerkrankungen, Carl-Neuberg-Str. 1, Hannover 30625, Germany |
| NZOZ Tri-medica U1. Rzgowska 281/289 Lodz 93–338, Poland |
| Saint-Petersburg State Budgetary Healthcare Institution “Children City Hospital #1”, Dialysis Dept, U1.avangardnaya, 14, St. Petersburg 198205, Russia |
| Semmelweis Egyetem Altalanos, Orvostudomanyi Kar, I.sz Germekgyogyaszati Klinika, Bokay Janos Utca 53, Budapest 1083, Hungary |
| Steve Biko Academic Hospital Dept. of Paediatrics, Room 71135, Cnr Malan and Steve Biko Street, Pretoria 0084, South Africa |
| Spitalul Clinic de Urgenta Pentru Copii – Louis Turcanu, Strada I Nemoianu No 2, Timisoara 300350, Romania |
| Spitalul Clinic de Urgenta Pentru, Copii Sf. Maria, Strada Vasile Lupu No 62, Iasi Romania |
| State Healthcare Institution of Moscow, Children City Clinical Hospital of saint Vladamir, Moscow, Russian Federation 107014 |
| Uniwersyteckie Centrum Kliniczne U1. Debinki 7, Gdansk 80–952, Poland |
| Uniwersytecki Dzieciecy Szpital Kliniczny, Im.1.zamenhoffa,klinika Pediatrii I Nefrologii, U1. Waszyngtona 17, Bialystok 15–274, Poland |
| Uniwerstyecki Szpital Dzieciecy W, Krakowie, Klinika Nefrologii, Dzieciecej Ze Stacja Dializ, U1. Wielicka 265, Krakow 30–663, Poland |
| Uniwerstyecki Szpital Kliniczny Im. Jana Mikulicza Radeckiego We Wroclawiu, Klinika U1. Borowska 213, Wroclaw 50–556, Poland |
