# Supplementary material for: An open-label phase 2 trial to assess the efficacy, safety and pharmacokinetics of lanthanum carbonate in hyperphosphatemic children and adolescents with chronic kidney disease undergoing dialysis
Source: BMC Nephrol. 2022 Mar 2;23:84. doi: 10.1186/s12882-022-02688-9 (PMC8892701; doi:10.1186/s12882-022-02688-9)
Supplement: Supplementary file 3 — Additional file 3: Table 1. Baseline demographics and characteristics of CC- and LC-treated patients, stratified by dialysis mode. [file 12882_2022_2688_MOESM3_ESM.docx]

**Additional table 2**

**An open-label phase 2 trial to assess the efficacy, safety and pharmacokinetics of lanthanum carbonate in hyperphosphatemic children and adolescents with chronic kidney disease undergoing dialysis**

Anna Wasilewska^1*^, RoseAnn Murray^2^, Aimee Sundberg^2^, Sharif Uddin^3^, Heinrich Achenbach^4^, Aleksey Shavkin^5^, Tamás Szabó^6^, Andrea Vergani^2^ and Obi Umeh^2^

*Correspondence: [anna.wasilewska@udsk.pl](mailto:anna.wasilewska@udsk.pl)

^1^Department of Pediatrics and Nephrology, Faculty of Medicine, Medical University of Bialystok, University Children’s Clinical Hospital of Bialystok, Waszyngtona, Bialystok, Poland
^2^Shire Human Genetic Therapies, Inc., a Takeda company, Cambridge, MA, USA
^3^Takeda Pharmaceuticals USA, Inc., Lexington, MA, USA

^4^Shire Human Genetic Therapies, Inc., a Takeda company, Zug, Switzerland ^5^Saint Petersburg State Budgetary Healthcare Institution, Children’s City Multidisciplinary Clinical Specialized Center of High Medical Technologies, Saint Petersburg, Russia
^6^Department of Pediatrics, Faculty of Medicine, University of Debrecen, Debrecen, Hungary

**Table 1** Baseline demographics and characteristics of CC- and LC-treated patients, stratified by dialysis mode

| **Characteristic** | **Part 1**  **(Safety analysis set 1)** | | **Part 2**  **(Safety analysis set 2)** | | | | **Parts 2 and 3 combined (Safety completer set)** | |
| --- | --- | --- | --- | --- | --- | --- | --- | --- |
|  | **LC**  **(n = 20)** | | **CC**  **(n = 53)** | | **LC**  **(n = 51)** | | **LC**  **(n = 46)** | |
|  | **Hemodialysis**  **(n = 12)** | **Peritoneal dialysis**  **(n = 8)** | **Hemodialysis**  **(n = 30)** | **Peritoneal dialysis**  **(n = 23)** | **Hemodialysis**  **(n = 29)** | **Peritoneal dialysis**  **(n = 22)** | **Hemodialysis**  **(n = 28)** | **Peritoneal dialysis**  **(n = 18)** |
| Age, years, mean (SD)^a^ | 13.4 (2.2) | 12.6 (3.3) | 13.1 (2.6) | 13.2 (3.0) | 13.7 (2.7) | 13.5 (2.7) | 14.0 (2.6) | 13.7 (2.8) |
| Age group, years, mean (SD) | | | | | | | | |
| <10^b^ | 0 (0.0) | 1 (12.5) | 1 (3.3) | 2 (8.7) | 1 (3.4) | 1 (4.5) | 1 (3.6) | 1 (5.6) |
| 10 to 11 | 2 (16.7) | 2 (25.0) | 6 (20.0) | 5 (21.7) | 4 (13.8) | 5 (22.7) | 2 (7.1) | 4 (22.2) |
| 12 to 17 | 10 (83.3) | 5 (62.5) | 23 (76.7) | 16 (69.6) | 24 (82.8) | 16 (72.7) | 25 (89.3) | 13 (72.2) |
| Sex, n (%) | | | | | | | | |
| Male | 5 (41.7) | 5 (62.5) | 17 (56.7) | 12 (52.2) | 18 (62.1) | 12 (54.5) | 17 (60.7) | 10 (55.6) |
| Female | 7 (58.3) | 3 (37.5) | 13 (43.3) | 11 (47.8) | 11 (37.9) | 10 (45.5) | 11 (39.3) | 8 (44.4) |
| Ethnicity, n (%) | | | | | | | | |
| Hispanic or Latino | 0 (0.0) | 0 (0.0) | 1 (3.3) | 4 (17.4) | 0 (0.0) | 4 (18.2) | 0 (0.0) | 4 (22.2) |
| Not Hispanic or Latino | 12 (100.0) | 8 (100.0) | 29 (96.7) | 19 (82.6) | 29 (100.0) | 18 (81.8) | 28 (100.0) | 14 (77.8) |
| Race, n (%) | | | | | | | | |
| White | 11 (91.7) | 8 (100.0) | 29 (96.7) | 23 (100.0) | 28 (96.6) | 22 (100.0) | 27 (96.4) | 18 (100.0) |
| Non-white | 1 (8.3) | 0 (0.0) | 1 (3.3) | 0 (0.0) | 1 (3.4) | 0 (0.0) | 1 (3.6) | 0 (0.0) |
| Weight, kg, mean (SD) | 43.2 (17.4) | 39.8 (16.0) | 36.9 (15.1) | 41.0 (15.2) | 41.1 (17.1) | 42.3 (14.5) | 41.8 (17.1) | 43.3 (15.0) |
| Height, cm, mean (SD) | 150.2 (16.4) | 147.7 (24.2) | 144.0 (19.1) | 147.8 (20.6) | 148.4 (19.7) | 150.0 (18.4) | 149.7 (19.5) | 150.3 (19.0) |
| Body mass index, kg/m^2^, mean (SD) | 18.5 (3.7) | 17.5 (3.0) | 17.3 (3.3) | 18.1 (3.0) | 18.2 (4.5) | 18.3 (3.1) | 18.3 (4.6) | 18.6 (3.0) |

Safety analysis set 1 included all patients who received at least one dose of LC in part 1 and attended at least one safety follow-up visit. Safety analysis set 2 included all patients who received at least one dose of CC or LC in part 2 and/or part 3 of the study and attended at least one safety follow-up visit. The safety completer set included all patients who received LC for at least 8 weeks in part 2 and/or part 3 of the study

^a^Age group was calculated as the difference between the date of birth and the date that informed consent/assent was received
^b^Patient demographics are presented for patients <10 years old who were included in the study before the protocol amendment, in which the inclusion criteria of 6 months to <18 years old was adjusted to 10 years to <18 years old

*CC* calcium carbonate; *LC* lanthanum carbonate; *SD* standard deviation
