# Supplementary material for: An open-label phase 2 trial to assess the efficacy, safety and pharmacokinetics of lanthanum carbonate in hyperphosphatemic children and adolescents with chronic kidney disease undergoing dialysis
Source: BMC Nephrol. 2022 Mar 2;23:84. doi: 10.1186/s12882-022-02688-9 (PMC8892701; doi:10.1186/s12882-022-02688-9)
Supplement: Supplementary file 4 — Additional file 4: Table 1. TEAEs considered related to study drug, experienced by ≥1 patient in ≥1 treatment arm. [file 12882_2022_2688_MOESM4_ESM.docx]

**Additional table 3**

**An open-label phase 2 trial to assess the efficacy, safety and pharmacokinetics of lanthanum carbonate in hyperphosphatemic children and adolescents with chronic kidney disease undergoing dialysis**

Anna Wasilewska^1*^, RoseAnn Murray^2^, Aimee Sundberg^2^, Sharif Uddin^3^, Heinrich Achenbach^4^, Aleksey Shavkin^5^, Tamás Szabó^6^, Andrea Vergani^2^ and Obi Umeh^2^

*Correspondence: [anna.wasilewska@udsk.pl](mailto:anna.wasilewska@udsk.pl)

^1^Department of Pediatrics and Nephrology, Faculty of Medicine, Medical University of Bialystok, University Children’s Clinical Hospital of Bialystok, Waszyngtona, Bialystok, Poland
^2^Shire Human Genetic Therapies, Inc., a Takeda company, Cambridge, MA, USA
^3^Takeda Pharmaceuticals USA, Inc., Lexington, MA, USA

^4^Shire Human Genetic Therapies, Inc., a Takeda company, Zug, Switzerland ^5^Saint Petersburg State Budgetary Healthcare Institution, Children’s City Multidisciplinary Clinical Specialized Center of High Medical Technologies, Saint Petersburg, Russia
^6^Department of Pediatrics, Faculty of Medicine, University of Debrecen, Debrecen, Hungary

**Table 1** TEAEs considered related to study drug, experienced by ≥1 patient in ≥1 treatment arm

| **Category of study drug-related TEAE** | **Safety analysis set 1 during part 1 of the study** | | **Safety analysis set 2 during part 2  of the study** | | | | **Safety analysis set 2 during part 2 and/or 3 of the study** | |
| --- | --- | --- | --- | --- | --- | --- | --- | --- |
|  | **LC**  **(n = 20)** | | **CC**  **(n = 53)** | | **LC**  **(n = 51)** | | **LC**  **(n = 52)** | |
|  | **n (%)** | **m** | **n** **(%)** | **m** | **n (%)** | **m** | **n (%)** | **m** |
| Any study drug-related TEAE | 2 (10.0) | 3 | 10 (18.9) | 16 | 8 (15.7) | 14 | 12 (23.1) | 21 |
| Eye disorders | | | | | | | | |
| Corneal degeneration | 0 (0.0) | 0 | 1 (1.9) | 1 | 0 (0.0) | 0 | 0 (0.0) | 0 |
| Gastrointestinal disorders | | | | | | | | |
| Vomiting | 1 (5.0) | 1 | 0 (0.0) | 0 | 1 (2.0) | 1 | 1 (1.9) | 1 |
| Nausea | 1 (5.0) | 1 | 1 (1.9) | 1 | 2 (3.9) | 2 | 2 (3.8) | 2 |
| Abdominal pain | 0 (0.0) | 0 | 0 (0.0) | 0 | 0 (0.0) | 0 | 1 (1.9) | 1 |
| Upper abdominal pain | 0 (0.0) | 0 | 0 (0.0) | 0 | 0 (0.0) | 0 | 1 (1.9) | 1 |
| Lip edema | 0 (0.0) | 0 | 0 (0.0) | 0 | 1 (2.0) | 2 | 1 (1.9) | 2 |
| Infections and infestations | | | | | | | | |
| Peritonitis | 0 (0.0) | 0 | 0 (0.0) | 0 | 1 (2.0) | 2 | 1 (1.9) | 2 |
| Appendicitis perforated | 0 (0.0) | 0 | 0 (0.0) | 0 | 1 (2.0) | 1 | 1 (1.9) | 1 |
| Metabolism and nutrition disorders | | | | | | | | |
| Hypercalcemia | 0 (0.0) | 0 | 8 (15.1) | 8 | 0 (0.0) | 0 | 0 (0.0) | 0 |
| Hypocalcemia | 0 (0.0) | 0 | 1 (1.9) | 1 | 0 (0.0) | 0 | 0 (0.0) | 0 |
| Hyperphosphatemia | 0 (0.0) | 0 | 1 (1.9) | 1 | 0 (0.0) | 0 | 0 (0.0) | 0 |
| Hypophosphatemia | 0 (0.0) | 0 | 2 (3.8) | 3 | 4 (7.8) | 5 | 6 (11.5) | 10 |
| Musculoskeletal and connective tissue disorders | | | | | | | | |
| Myalgia | 0 (0.0) | 0 | 1 (1.9) | 1 | 0 (0.0) | 0 | 0 (0.0) | 0 |
| Nervous system disorders | | | | | | | | |
| Somnolence | 1 (5.0) | 1 | 0 (0.0) | 0 | 0 (0.0) | 0 | 0 (0.0) | 0 |
| Skin and subcutaneous tissue disorders | | | | | | | | |
| Rash | 0 (0.0) | 0 | 0 (0.0) | 0 | 1 (2.0) | 1 | 1 (1.9) | 1 |

TEAEs were categorized by the treatment most recently received by the patient and patients were counted once per category, per treatment group; adverse events were considered TEAEs if they occurred in the 3 weeks after the most recent dose of study drug within the relevant part of the study. Reported TEAEs are stratified by treatment group and by system organ class and preferred term

Safety analysis set 1 included all patients who received at least one dose of LC in part 1 and attended at least one safety follow-up visit. Safety analysis set 2 included all patients who received at least one dose of CC or LC in part 2 and/or part 3 of the study and attended at least one safety follow-up visit.

*CC* calcium carbonate; *LC* lanthanum carbonate; *m* the number of events experienced; *TEAE* treatment-emergent adverse event
