# Supplementary material for: An open-label phase 2 trial to assess the efficacy, safety and pharmacokinetics of lanthanum carbonate in hyperphosphatemic children and adolescents with chronic kidney disease undergoing dialysis
Source: BMC Nephrol. 2022 Mar 2;23:84. doi: 10.1186/s12882-022-02688-9 (PMC8892701; doi:10.1186/s12882-022-02688-9)
Supplement: Supplementary file 5 — Additional file 5: Table 1. Change from baseline in biochemical bone markers for patients in part 2 of the studya. [file 12882_2022_2688_MOESM5_ESM.docx]

**Additional table 4**

**An open-label phase 2 trial to assess the efficacy, safety and pharmacokinetics of lanthanum carbonate in hyperphosphatemic children and adolescents with chronic kidney disease undergoing dialysis**

Anna Wasilewska^1*^, RoseAnn Murray^2^, Aimee Sundberg^2^, Sharif Uddin^3^, Heinrich Achenbach^4^, Aleksey Shavkin^5^, Tamás Szabó^6^, Andrea Vergani^2^ and Obi Umeh^2^

*Correspondence: [anna.wasilewska@udsk.pl](mailto:anna.wasilewska@udsk.pl)

^1^Department of Pediatrics and Nephrology, Faculty of Medicine, Medical University of Bialystok, University Children’s Clinical Hospital of Bialystok, Waszyngtona, Bialystok, Poland
^2^Shire Human Genetic Therapies, Inc., a Takeda company, Cambridge, MA, USA
^3^Takeda Pharmaceuticals USA, Inc., Lexington, MA, USA

^4^Shire Human Genetic Therapies, Inc., a Takeda company, Zug, Switzerland ^5^Saint Petersburg State Budgetary Healthcare Institution, Children’s City Multidisciplinary Clinical Specialized Center of High Medical Technologies, Saint Petersburg, Russia
^6^Department of Pediatrics, Faculty of Medicine, University of Debrecen, Debrecen, Hungary

**Table 1** Change from baseline in biochemical bone markers for patients in part 2 of the study^a^

| **Biochemical bone marker** | **CC**  **(n = 17)** | **LC**  **(n = 17)** |
| --- | --- | --- |
| Bone alkaline phosphatase, μg/L | | |
| Baseline | 72.3 (15.1) | 72.0 (13.8) |
| Week 8 of treatment^b^ | 73.2 (14.4) | 92.2 (21.4) |
| Change from baseline^b^ | −0.8 (8.5) | 31.9 (16.3) |
| Osteocalcin, μg/L | | |
| Baseline | 455.2 (32.2) | 451.8 (37.8) |
| Week 8 of treatment^b^ | 454.3 (35.2) | 458.9 (37.2) |
| Change from baseline^b^ | 8.2 (20.2) | 10.3 (21.6) |
| Tartrate-resistant acid phosphatase, U/L | | |
| Baseline | 16.5 (2.1) | 16.0 (1.8) |
| Week 8 of treatment^b^ | 15.2 (1.6) | 16.8 (1.4) |
| Change from baseline^b^ | −1.7 (1.1) | 2.0 (1.2) |
| Fibroblast growth factor-23, RU/L | | |
| Baseline | 7018.2 (2922.8) | 12101.9 (6666.7) |
| Week 8 of treatment^b^ | 12574.6 (7079.8) | 5515.0 (2336.6) |
| Change from baseline^b^ | 5130.3 (5232.2) | −8162.4 (5274.6) |
| Parathyroid hormone, pmol/L | | |
| Baseline | 35.5 (5.8) | 30.6 (6.4) |
| Week 8 of treatment^b^ | 28.5 (8.5) | 50.1 (11.8) |
| Change from baseline^b^ | −7.1 (9.8) | 16.3 (12.5) |
| Sclerostin, μg/L | | |
| Baseline^c^ | 1.0 (0.1) | 1.1 (0.1) |
| Week 8 of treatment^d^ | 1.2 (0.2) | 1.0 (0.1) |
| Change from baseline^d^ | 0.1 (0.1) | −0.1 (0.1) |
| Fetuin-A, g/L | | |
| Baseline^c^ | 0.46 (0.04) | 0.42 (0.04) |
| Week 8 of treatment^d^ | 0.44 (0.04) | 0.46 (0.03) |
| Change from baseline^d^ | −0.01 (0.03) | 0.04 (0.04) |

^a^All data are presented as mean (SEM) for patients from per-protocol set 1, which included all patients who received CC for 8 weeks during part 2, followed by a washout period and then LC for at least 8 weeks during part 2 and/or 3, and who had serum phosphorus data available for analysis. Only patients who had serum phosphorus levels above the age-specific KDOQI targets at study entry and between the CC and LC treatment regimens or the visits during the washout before either part 1 or part 2 were included in this set. One patient did not complete the 8 weeks of CC treatment and progressed straight to the 8 weeks of LC treatment. ^b^CC, n = 16; LC, n = 15. ^c^CC, n = 13; LC, n = 13. ^d^CC, n = 12; LC, n = 13.

*CC* calcium carbonate; *LC* lanthanum carbonate; *KDOQI* Kidney Disease Outcomes Quality Initiative; *SEM*standard error of the mean
