# Supplementary material for: An open-label phase 2 trial to assess the efficacy, safety and pharmacokinetics of lanthanum carbonate in hyperphosphatemic children and adolescents with chronic kidney disease undergoing dialysis
Source: BMC Nephrol. 2022 Mar 2;23:84. doi: 10.1186/s12882-022-02688-9 (PMC8892701; doi:10.1186/s12882-022-02688-9)
Supplement: Supplementary file 6 — Additional file 6: Table 1. Frequently reported TEAEs (≥10% of patients in ≥1 treatment arm) in parts 2 and 3 of the study, stratified by dialysis modality. [file 12882_2022_2688_MOESM6_ESM.docx]

**Additional table 5**

**An open-label phase 2 trial to assess the efficacy, safety and pharmacokinetics of lanthanum carbonate in hyperphosphatemic children and adolescents with chronic kidney disease undergoing dialysis**

Anna Wasilewska^1*^, RoseAnn Murray^2^, Aimee Sundberg^2^, Sharif Uddin^3^, Heinrich Achenbach^4^, Aleksey Shavkin^5^, Tamás Szabó^6^, Andrea Vergani^2^ and Obi Umeh^2^

*Correspondence: [anna.wasilewska@udsk.pl](mailto:anna.wasilewska@udsk.pl)

^1^Department of Pediatrics and Nephrology, Faculty of Medicine, Medical University of Bialystok, University Children’s Clinical Hospital of Bialystok, Waszyngtona, Bialystok, Poland
^2^Shire Human Genetic Therapies, Inc., a Takeda company, Cambridge, MA, USA
^3^Takeda Pharmaceuticals USA, Inc., Lexington, MA, USA

^4^Shire Human Genetic Therapies, Inc., a Takeda company, Zug, Switzerland ^5^Saint Petersburg State Budgetary Healthcare Institution, Children’s City Multidisciplinary Clinical Specialized Center of High Medical Technologies, Saint Petersburg, Russia
^6^Department of Pediatrics, Faculty of Medicine, University of Debrecen, Debrecen, Hungary

**Table 1** Frequently reported TEAEs (≥10% of patients in ≥1 treatment arm) in parts 2 and 3 of the study, stratified by dialysis modality

| **Category of TEAE** | **Safety analysis set 2 during  part 2 of the study** | | | | | | | | | **Safety analysis set 2 during part 2 and/or part 3 of the study** | | | |
| --- | --- | --- | --- | --- | --- | --- | --- | --- | --- | --- | --- | --- | --- |
|  | **CC**  **(n = 53)** | | | | | **LC**  **(n = 51)** | | | | **LC**  **(n = 52)** | | | |
|  | **Hemodialysis**  **(n = 30)** | | **Peritoneal dialysis**  **(n = 23)** | | | **Hemodialysis**  **(n = 29)** | | **Peritoneal dialysis**  **(n = 22)** | | **Hemodialysis**  **(n = 30)** | | **Peritoneal dialysis**  **(n = 22)** | |
|  | **n** **(%)** | **m** | **n** **(%)** | | **m** | **n (%)** | **m** | **n (%)** | **m** | **n (%)** | **m** | **n (%)** | **m** |
| Any | 17 (56.7) | 41 | 11 (47.8) | 22 | | 17 (58.6) | 40 | 11 (50.0) | 16 | 24 (80.0) | 85 | 17 (77.3) | 39 |
| Serious | 7 (23.3) | 8 | 2 (8.7) | 2 | | 8 (27.6) | 12 | 3 (13.6) | 5 | 13 (43.3) | 21 | 6 (27.3) | 8 |
| Related to study drug | 7 (23.3) | 10 | 3 (13.0) | 6 | | 7 (24.1) | 11 | 1 (4.5) | 3 | 10 (33.3) | 17 | 2 (9.1) | 4 |
| Leading to study  withdrawal | 2 (6.7) | 2 | 0 (0.0) | 0 | | 2 (6.9) | 3 | 1 (4.5) | 3 | 2 (6.7) | 3 | 1 (4.5) | 3 |
| Leading to death | 0 (0.0) | 0 | 0 (0.0) | 0 | | 0 (0.0) | 0 | 0 (0.0) | 0 | 0 (0.0) | 0 | 0 (0.0) | 0 |
| TEAEs occurring in ≥3% of patients in ≥1 treatment arm as presented in Table 5 | | | | | | | | | | | | | |
| Blood and lymphatic system disorders | | | | | | | | | | | | | |
| Anemia | 0 (0.0) | 0 | 0 (0.0) | 0 | | 0 (0.0) | 0 | 0 (0.0) | 0 | 2 (6.7) | 2 | (0.0) | 0 |
| Gastrointestinal disorders | | | | | | | | | | | | | |
| Vomiting | 0 (0.0) | 0 | 1 (4.3) | 2 | | 1 (3.4) | 1 | 1 (4.5) | 1 | 3 (10.0) | 4 | 3 (13.6) | 3 |
| Nausea | 1 (3.3) | 1 | 1 (4.3) | 1 | | 2 (6.9) | 3 | 0 (0.0) | 0 | 2 (6.7) | 3 | 2 (9.1) | 3 |
| Abdominal pain | 0 (0.0) | 0 | 1 (4.3) | 1 | | 0 (0.0) | 0 | 0 (0.0) | 0 | 0 (0.0) | 0 | 2 (9.1) | 3 |
| Diarrhea | 2 (6.7) | 3 | 0 (0.0) | 0 | | 0 (0.0) | 0 | 0 (0.0) | 0 | 0 (0.0) | 0 | 0 (0.0) | 0 |
| General disorders and administration site conditions | | | | | | | | | | | | | |
| Pyrexia | 0 (0.0) | 0 | 0 (0.0) | 0 | | 1 (3.4) | 1 | 0 (0.0) | 0 | 2 (6.7) | 3 | 0 (0.0) | 0 |
| Infections/infestations | | | | | | | | | | | | | |
| Peritonitis | 0 (0.0) | 0 | 0 (0.0) | 0 | | 0 (0.0) | 0 | 3 (13.6) | 4 | 0 (0.0) | 0 | 5 (22.7) | 6 |
| Upper respiratory tract infection | 0 (0.0) | 0 | 0 (0.0) | 0 | | 0 (0.0) | 0 | 3 (13.6) | 3 | 1 (3.3) | 1 | 4 (18.2) | 4 |
| Nasopharyngitis | 1 (3.3) | 1 | 1 (4.3) | 1 | | 0 (0.0) | 0 | 0 (0.0) | 0 | 1 (3.3) | 2 | 1 (4.5) | 1 |
| Device related infection | 0 (0.0) | 0 | 0 (0.0) | 0 | | 2 (6.9) | 2 | 0 (0.0) | 0 | 2 (6.7) | 3 | 0 (0.0) | 0 |
| Respiratory tract infection | 2 (6.7) | 4 | 1 (4.3) | 1 | | 0 (0.0) | 0 | 0 (0.0) | 0 | 1 (3.3) | 1 | 1 (4.5) | 1 |
| Hordeolum | 0 (0.0) | 0 | 0 (0.0) | 0 | | 0 (0.0) | 0 | 1 (4.5) | 1 | 1 (3.3) | 1 | 1 (4.5) | 1 |
| Influenza | 0 (0.0) | 0 | 2 (8.7) | 2 | | 0 (0.0) | 0 | 0 (0.0) | 0 | 1 (3.3) | 1 | 0 (0.0) | 0 |
| Metabolism and nutrition disorders | | | | | | | | | | | | | |
| Hypercalcemia | 6 (20.0) | 6 | 3 (13.0) | 3 | | 3 (10.3) | 3 | 1 (4.5) | 1 | 4 (13.3) | 8 | 1 (4.5) | 1 |
| Hypocalcemia | 3 (10.0) | 3 | 0 (0.0) | 0 | | 1 (3.4) | 1 | 0 (0.0) | 0 | 2 (6.7) | 4 | 1 (4.5) | 2 |
| Hyperkalemia | 1 (3.3) | 1 | 0 (0.0) | 0 | | 0 (0.0) | 0 | 0 (0.0) | 0 | 0 (0.0) | 0 | 2 (9.1) | 2 |
| Hyperphosphatemia | 2 (6.7) | 2 | 0 (0.0) | 0 | | 0 (0.0) | 0 | 0 (0.0) | 0 | 0 (0.0) | 0 | 0 (0.0) | 0 |
| Hypophosphatemia | 2 (6.7) | 3 | 1 (4.3) | 1 | | 4 (13.8) | 5 | 0 (0.0) | 0 | 6 (20.0) | 10 | 0 (0.0) | 0 |
| Vascular disorders | | | | | | | | | | | | | |
| Hypotension | 0 (0.0) | 0 | 0 (0.0) | 0 | | 1 (3.4) | 3 | 1 (4.5) | 1 | 1 (3.3) | 3 | 0 (0.0) | 0 |
| Hypertension^d^ | 2 (6.7) | 2 | 1 (4.3) | 1 | | 1 (3.4) | 1 | 0 (0.0) | 0 | 1 (3.3) | 1 | 0 (0.0) | 0 |

Safety analysis set 2 included all patients who received at least one dose of CC or LC in part 2 and/or part 3 of the study and attended at least one follow-up visit. Reported TEAEs are stratified by treatment group, type of dialysis modality and preferred term. Most TEAEs were considered by the investigator to be mild or moderate in severity, unless otherwise indicated.

*CC* calcium carbonate; *LC* lanthanum carbonate; *m* the number of events experienced; *TEAE* treatment-emergent adverse event
